# Supplementary material for: Comprehensive Annotation and Functional Exploration of MicroRNAs in Lettuce
Source: Front Plant Sci. 2021 Dec 24;12:781836. doi: 10.3389/fpls.2021.781836 (PMC8739914; doi:10.3389/fpls.2021.781836)
Supplement: Supplementary file 2 [file Data_Sheet_8.ZIP › GSM2230749.plot/Lsa-miR396d_Lsat_1_v5_gn_9_63600.1_701_TPlot.pdf]

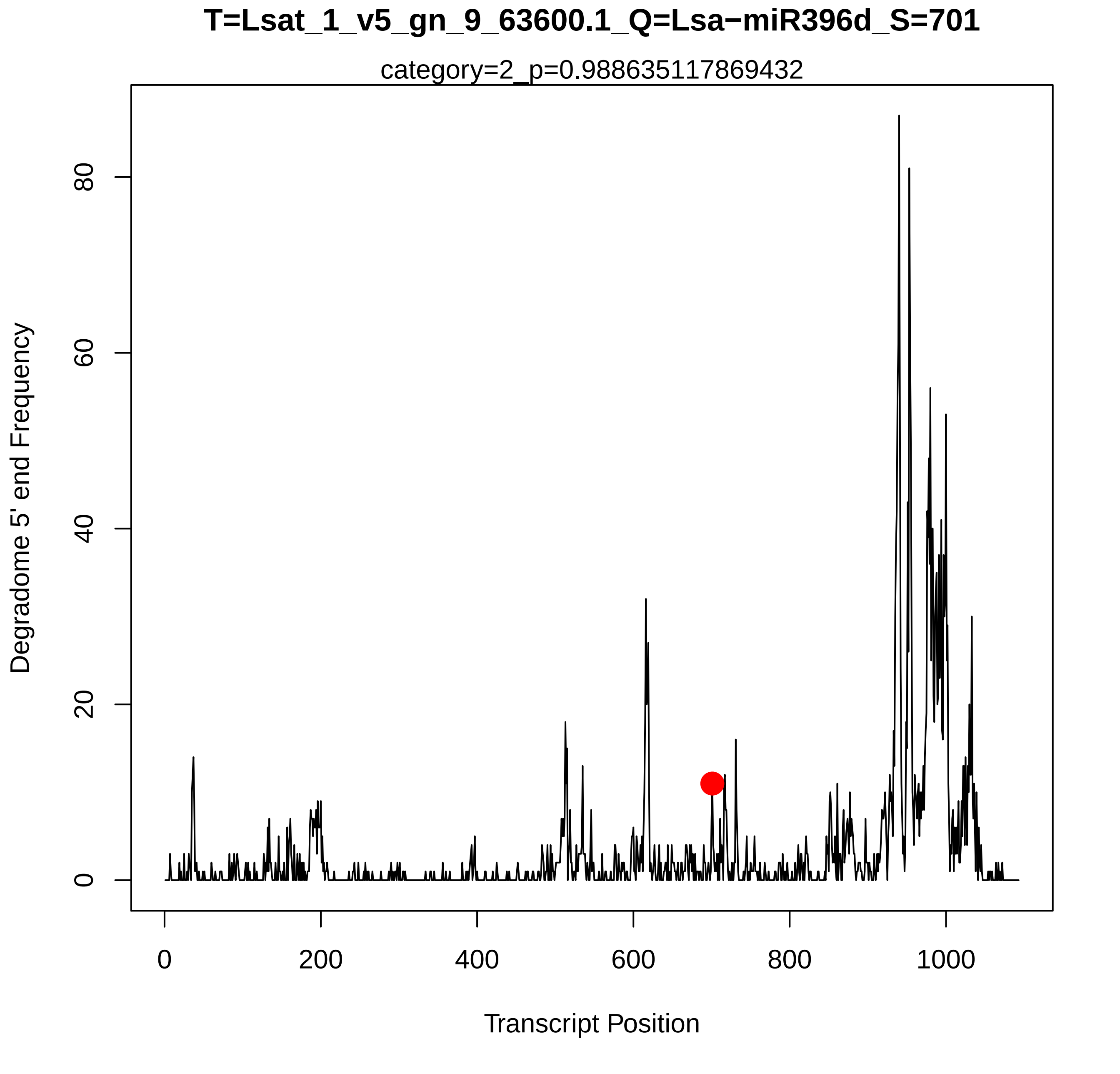

T=Lsat_1_v5_gn_9_63600.1_Q=Lsa−miR396d_S=701
category=2_p=0.988635117869432
Degradome 5' end Frequency
0
20
40
60
80
0
200
400
600
800
1000
Transcript Position
